# Liver disease in management and outcomes of European and Asian patients with atrial fibrillation: A report from two observational prospective registries

**DOI:** 10.1111/eci.70193

**Published:** 2026-03-26

**Authors:** Davide Antonio Mei, Tommaso Bucci, Giulio Francesco Romiti, Bernadette Corica, Alena Shantsila, Hung‐Fat Tse, Giuseppe Boriani, Tze‐Fan Chao, Marco Proietti, Gregory Y. H. Lip

**Affiliations:** ^1^ Liverpool Centre for Cardiovascular Science at University of Liverpool Liverpool John Moores University and Liverpool Heart & Chest Hospital Liverpool UK; ^2^ Cardiology Division, Department of Biomedical, Metabolic and Neural Sciences Italy University of Modena and Reggio Emilia, Policlinico di Modena Modena Italy; ^3^ Clinical and Experimental Medicine PhD Program University of Modena and Reggio Emilia Modena Italy; ^4^ Department of Translational and Precision Medicine Sapienza – University of Rome Rome Italy; ^5^ Division of Cardiology, Department of Medicine, School of Clinical Medicine Queen Mary Hospital, the University of Hong Kong Hong Kong SAR China; ^6^ Division of Cardiology, Department of Medicine Taipei Veterans General Hospital Taipei Taiwan; ^7^ Institute of Clinical Medicine, and Cardiovascular Research Center National Yang Ming Chiao Tung University Taipei Taiwan; ^8^ Department of Clinical Sciences and Community Health University of Milan Milan Italy; ^9^ Division of Subacute Care IRCCS Istituti Clinici Scientifici Maugeri Milan Italy; ^10^ Department of Clinical Medicine Aalborg University Aalborg Denmark; ^11^ Department of Cardiology, Lipidology and Internal Medicine with Intensive Coronary Care Unit Medical University of Bialystok Bialystok Poland

**Keywords:** atrial fibrillation, liver disease, oral anticoagulation, outcomes

## Abstract

**Background:**

In patients with atrial fibrillation (AF), the impact of liver disease (LD) on oral anticoagulant (OAC) prescription and outcomes remains unclear, as well as possible differences between European and Asian populations.

**Aim:**

To examine the impact of LD on OAC prescriptions and risks of adverse outcomes in a large cohort of European and Asian AF patients.

**Methods:**

AF patients were derived from two large observational registries held in Europe and Asia. OAC prescription and risk of outcomes were analysed according to LD at baseline. The primary outcome was the composite of all‐cause death and major adverse cardiovascular events (MACEs). Logistic regression assessed associations with OAC prescription, and Cox regression analyses evaluated risks of outcomes. Interaction analyses were performed between European and Asian patients.

**Results:**

Among 15,681 patients (mean age 68.4 ± 10.7 years; 37.1% female), 517 (3.3%) had LD. The OAC prescription rate was similar among European and Asian individuals (6.8% vs. 82.9%, *p* = .113). After adjustments, LD was associated with lower OAC prescription (OR .67, 95% CI .53–.84), with a greater reduction in European than in Asian patients (*p*_int_ = .015). LD was associated with a higher risk of the composite outcome (HR 1.42, 95% CI 1.11–1.81) and MACEs (HR 1.47, 95% CI 1.07–2.02), with no significant European versus Asian differences (*p*_int_ = .631). Among LD patients, those not prescribed OAC had a higher MACE risk compared with those prescribed OAC (*p*_int_ = .050), with no differences in major bleeding.

**Conclusions:**

In AF, LD is associated with reduced OAC prescription, especially in Europe, and a higher risk of adverse outcomes, particularly in patients not receiving OAC, with no significant differences between European and Asian cohorts.

## Graphical abstract

In this large European–Asian cohort of patients with atrial fibrillation, liver disease was linked to lower oral anticoagulant use—particularly in Europe—and to higher risks of mortality and cardiovascular events. These findings highlight the adverse impact of liver disease, especially when oral anticoagulation is withheld.

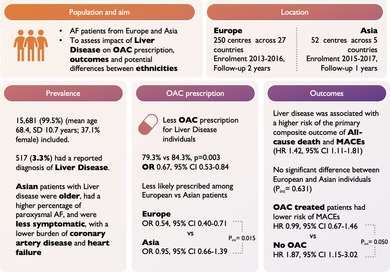

## 1 INTRODUCTION

Liver disease (LD) is a major global health issue and is responsible for more than 1 million deaths every year,^1^ with an impact on healthcare resources use and mortality projected to triple by 2030.^2^ Similarly, atrial fibrillation (AF) is associated with significant morbidity and mortality, as well as hospitalisations and healthcare costs.^3^, ^4^ LD patients are at increased risk of developing AF, independently of other traditional risk factors.^5^, ^6^ Therefore, the increasing number of patients with both diseases will require a holistic approach to optimisation of their management.

Among AF patients, oral anticoagulation (OAC) is recommended by guidelines with a preference for non‐vitamin K antagonist oral anticoagulants (NOACs) over vitamin K antagonists (VKAs).^7^, ^8^ However, in past years, undertreatment with OAC therapy has been described in patients with concomitant LD.^9^ The exclusion of patients with LD from the clinical trials for the approval of NOACs and the fear of a higher risk of bleeding events may in part explain these findings.

Available data suggest that LD is a condition characterized by both an increased risk of thromboembolic and bleeding events,^10^ thus possibly modulating the risk of outcomes in AF patients. However, how LD may influence the clinical history of AF patients across different countries and ethnicities remains not entirely understood.

Therefore, we conducted this analysis to investigate the impact of LD on the prescription of OAC and the risks of adverse outcomes in a large cohort of European and Asian AF patients, while also examining possible differences between ethnicities.

## 2 METHODS

### 2.1 Study design and cohort

We studied AF patients enrolled in two large prospective observational registries held in Europe and Asia. Details on the studies design, baseline characteristics, and primary results have been previously published.^11^, ^12^ Briefly, both registries enrolled consecutive patients with an ECG‐documented episode of AF in the 12 months before inclusion. All patients were >18 years old and provided informed consent.

For the European registry, patients were enrolled in 250 participating centres across 27 countries. The enrolment was performed between October 2013 and September 2016, with a 2‐year follow‐up until September 2018. The study protocol was approved for each country and for each enrolling site by the National Coordinators' main institutions. The study was performed according to the European Union Note for Guidance on Good Clinical Practice CPMP/ECH/135/95 and the Declaration of Helsinki.

The Asian registry enrolled patients in 52 centres across five countries. It was established in late 2015 and patients were enrolled until early 2017, with a 1‐year follow‐up observation. The study protocol was approved by each institution's local ethics committee. The use of a shared study design, identical inclusion criteria, and the same electronic case report form (eCRF) across the two registries ensured a high level of methodological consistency. This approach minimized potential sources of bias related to patient selection, variable definition and data collection.

On this basis, the two datasets were merged to allow comparative analyses between European and Asian AF populations. Merging the databases enabled the evaluation of potential interactions between geographical region (Europe vs. Asia) and clinical management strategies or outcomes, as previously reported in other studies from the same registries.^13^, ^14^, ^15^ As this was a prospective observational registry‐based analysis, no formal a priori sample size or power calculation was performed; the study population was defined by consecutive patient enrolment during the prespecified study periods.

For this analysis, we included AF patients with available data on the history of LD.

LD was identified by investigators at site level at the time of enrolment, based on clinical evaluation and review of available medical records. The diagnosis of LD was established according to routine clinical practice, considering a documented history of chronic LD, including chronic viral hepatitis, nonalcoholic or alcoholic liver disease, autoimmune liver disorders or other causes of chronic hepatic dysfunction. Evidence supporting the diagnosis could include previous physician‐reported diagnoses, imaging or clinical findings consistent with chronic LD or cirrhosis or persistently abnormal liver function tests suggestive of chronic hepatic impairment.

This assessment was performed by any physician involved in the patient's care. No standardized diagnostic criteria, centralized adjudication, or systematic collection of data regarding liver disease aetiology, stage, or severity were implemented in the study protocol. For the purpose of the present analysis, patients were categorized into two groups: (i) ‘No Liver Disease’ and (ii) ‘Liver Disease’.

### 2.2 Pharmacological treatments

At baseline, data regarding pharmacological treatment at discharge/after consultation were collected by the investigator. We compared the prescription of several pharmacological treatments for patients with and without a diagnosis of LD.

Pharmacological treatments evaluated in this analysis were as follows: (i) OAC therapy (VKAs or NOACs); (ii) NOACs versus VKAs; (iii) class IC antiarrhythmic drugs (AADs [defined as treatment with Flecainide or Propafenone]); class III AADs (defined as treatment with Amiodarone or Dronedarone or Sotalol); Any AADs (defined as class IC or III AADs); (vi) Beta‐blockers; (vii) Nondihydropyridines calcium channel blockers (Non‐DHP CCB [defined as therapy with Diltiazem or Verapamil]); (viii) Digoxin; (ix) Any rate control therapy (defined as treatment with Beta‐blocker or Non‐DHP CCB or Digoxin). We also evaluated possible differences in the prescription of such therapies between the two registries.

### 2.3 Follow‐up and adverse outcomes

As per the original studies design, patients enrolled in the European registry were followed up for 2 years, while those in the Asian registry for 1 year. Occurrence of major adverse events was collected by the investigators during observation. Adverse events considered were as follows: (i) All‐cause death; (ii) Cardiovascular (CV) death; (iii) Any acute coronary syndrome (ACS); (iv) any thromboembolic (TE) events (defined as a composite of stroke, transient ischemic attack [TIA] and peripheral embolism); (v) Major Bleeding (defined as a composite of intracranial or extracranial major bleeding).

For this analysis, our primary endpoint was a composite outcome of all‐cause death, any ACS and any TE. As secondary endpoints, we evaluated: (i) All‐cause death; (ii) a composite endpoint of major adverse cardiovascular events (MACEs) (defined as CV‐death, any ACS or any TE); (iii) major bleeding. We also compared the differential risk of adverse outcomes in the two registries, according to the use of OAC, and for NOACs versus VKAs users.

### 2.4 Statistical analysis

Continuous variables are reported as median and interquartile range (IQR). Comparison between groups has been performed using the Kruskal–Wallis test or the Mann–Whitney U test as appropriate. Categorical variables are reported as counts and percentages and compared using the chi‐square test or the Fisher's exact *t*‐test (if expected cell count was less than 5).

Multivariable logistic regressions were used to evaluate the probability of prescription of pharmacological treatments according to the presence of LD. Interaction models were used to compare the results among the two registries. Results are reported as Odds Ratio (OR) with 95% Confidence Interval (CI).

Cumulative survival curves for each endpoint among the two groups were plotted using Kaplan–Meier curves and tested for difference with the log‐rank test. Cox regressions were used to investigate the relationship between LD and adverse outcomes. We performed a multivariable analysis adjusting the model for: age, gender, type of AF, CHA_2_DS_2_‐VASc score (HAS‐BLED score for major bleeding), EHRA score and any OAC therapy. Interaction analyses were used to investigate the effect of LD on modulating the risk of adverse outcomes in the prespecified subgroups. The multivariable models were not adjusted for LD severity or liver function since these data were not systematically collected.

Results are reported as Hazard Ratio (HR) with 95% Confidence Interval (CI). A two‐sided *p*‐value <.05 was considered statistically significant. All analyses were performed using R 4.0.3 (R Core Team 2020, Vienna, Austria).

## 3 RESULTS

Among the 15,762 AF patients available after merging the two datasets, 15,681 (99.5%) (mean age 68.4, SD 10.7 years; 37.1% female) had complete data regarding LD and were included in the analysis. Among these individuals, 517 (3.3%) had a reported diagnosis of LD.

Table 1 shows the baseline characteristics of the two cohorts. Overall, AF patients with LD were more likely to have permanent AF, be symptomatic, and have higher thromboembolic and bleeding risks, with a higher prevalence of several concomitant conditions.

**TABLE 1:** Baseline characteristics of patients with and without liver disease.

	No liver disease *N* = 15,164 (96.7%)	Liver disease *N* = 517 (3.3%)	*p*
Age (years) (median [IQR])	70.00 [62.00, 77.00]	69.00 [62.00, 75.00]	.068
Female, *n* (%)	5879 (38.8)	192 (37.1)	.482
BMI (median [IQR])	26.70 [24.00, 30.10]	26.40 [23.50, 29.40]	.049
Cardiovascular diseases			
Hypertension, *n* (%)	9271 (61.6)	327 (63.9)	.324
Diabetes mellitus, *n* (%)	3459 (23.0)	175 (34.2)	**<.001**
Dyslipidemia, *n* (%)	5838 (39.8)	249 (49.7)	**<.001**
Coronary artery disease, *n* (%)	3774 (26.1)	140 (29.0)	.159
Heart Failure, *n* (%)	5027 (33.4)	258 (51.1)	**<.001**
Previous TE events, *n* (%)	1714 (11.4)	69 (13.5)	.157
Peripheral vascular disease, *n* (%)	885 (5.9)	48 (9.6)	.**001**
Comorbidities			
CKD, *n* (%)	1621 (10.7)	106 (20.7)	**<.001**
CrCl CG (median [IQR])	71.54 [52.78, 93.06]	66.28 [48.72, 87.57]	.**003**
Malignancy (current+prior), *n* (%)	1136 (7.5)	51 (10.1)	.**040**
Previous hemorrhagic events, *n* (%)	880 (5.9)	45 (8.9)	.**006**
Anaemia, *n* (%)	863 (5.7)	72 (14.0)	**<.001**
AF type, *n* (%)			
First diagnosed	2011 (13.4)	55 (10.8)	**<.001**
Paroxysmal	4658 (31.1)	126 (24.7)	
Persistent	3101 (20.7)	98 (19.2)	
Long‐standing persistent	894 (6.0)	36 (7.1)	
Permanent	4303 (28.7)	195 (38.2)	
CHA_2_DS_2_‐VASc (median [IQR])	3.00 [2.00, 4.00]	3.00 [2.00, 5.00]	**<.001**
HAS‐BLED (median [IQR])	1.00 [1.00, 2.00]	3.00 [2.00, 3.00]	**<.001**
EHRA score 3–4, *n* (%)	2331 (15.4)	101 (19.5)	.**012**

*Note:* Bold values indicate the *p* values that were significant.

Abbreviations: CG, Cockcroft‐Gault; CKD, chronic kidney disease; CrCl, creatinine clearance; IQR, interquartile range; TE, thromboembolic events.

Table S1 presents the baseline characteristics of patients with LD from the two registries. Compared to their European counterparts, Asian AF patients with LD were older, had a higher percentage of paroxysmal AF, and were less symptomatic, with a lower burden of coronary artery disease and heart failure. Similar thromboembolic and bleeding risks were observed among LD patients across both registries.

### 3.1 Pharmacological treatments

**FIGURE 1:**
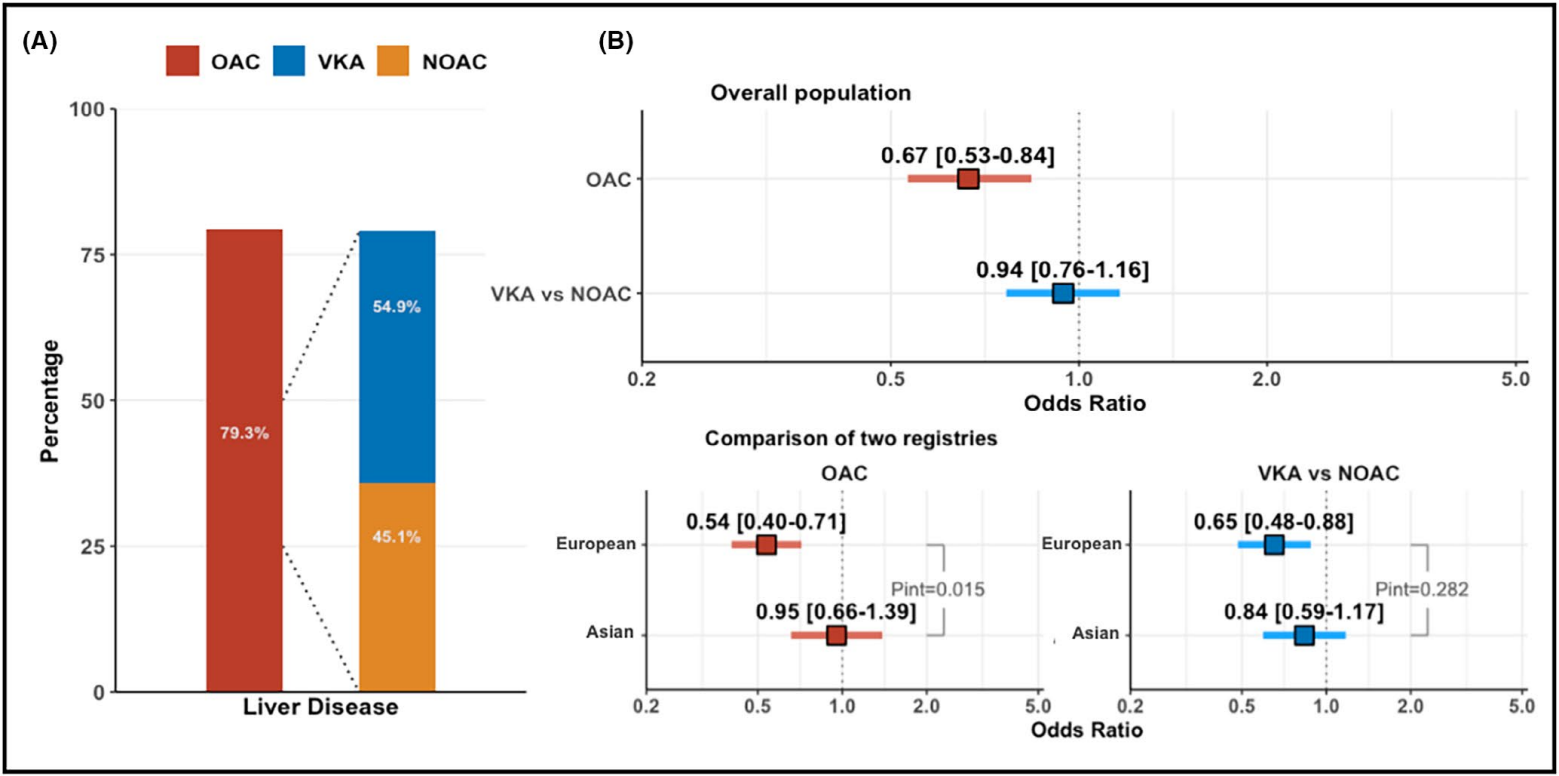
(A) Percentage of patients with LD prescribed with OAC and distribution of VKA and NOAC therapy in the overall population. (B) Probability of prescription of OAC and NOAC versus VKA in the overall population and in the two registries. Association between liver disease and oral anticoagulant prescription. Squares represent the point estimates expressed as odds ratios, and horizontal lines indicate 95% confidence intervals. NOAC, non‐vitamin K oral anticoagulant; OAC, oral anticoagulant; VKA, vitamin K anticoagulant.

Patients with LD were less prescribed an OAC therapy (84.3% vs. 79.3%, *p* = .003, Table S2); among them, 54.9% were prescribed a VKA and 45.1% a NOAC (Figure 1). They were treated less often with NOAC and AADs and more frequently prescribed rate control treatments (Table S2). In the multivariate logistic regression, LD was associated with a lower prescription of OAC (OR .67, 95% CI .53–.84 [Figure 1]) and Any AADs (OR .79, 95% CI .62–.99) (Figure S1). LD patients were more likely to be prescribed Digoxin (OR 1.81, 95% CI 1.45–2.25) (Figure S1), whereas no significant differences were found for other rate control drugs considered in the analysis.

Table S3 shows the pharmacological management of patients with LD in the two registries. European and Asian patients with LD had similar OAC therapy prescription (76.8% vs. 82.9%, *p* = .113). Asian patients were less likely to be prescribed AADs (25.1% vs. 13.4%, *p* = .002) and had any rate control therapy (80.9% vs. 69.6%, *p* = .005). After adjustments, a significant interaction effect was found between LD and registries on modulating the probability of prescription of OAC (*p*_int_ = .015): in particular, European patients had a lower likelihood of being prescribed OAC (OR .54, 95% CI .40–.71) compared to Asian patients (OR .95, 95% CI .66–1.39). No significant interaction effect was found between LD and the prescription of NOACs vs. VKAs (Figure 1).

Figure S1 shows the association between LD and other pharmacological treatments in the two registries. There was a significant interaction effect between LD and the registries on the prescription of class III AADs and Digoxin. In particular, Asian patients with LD had a lower probability of being prescribed a class III AAD. (*p*_int_ = .030) and Digoxin compared to Europeans (*p*_int_ = .008) (Figure S1).

### 3.2 Follow‐up and adverse outcomes

**FIGURE 2:**
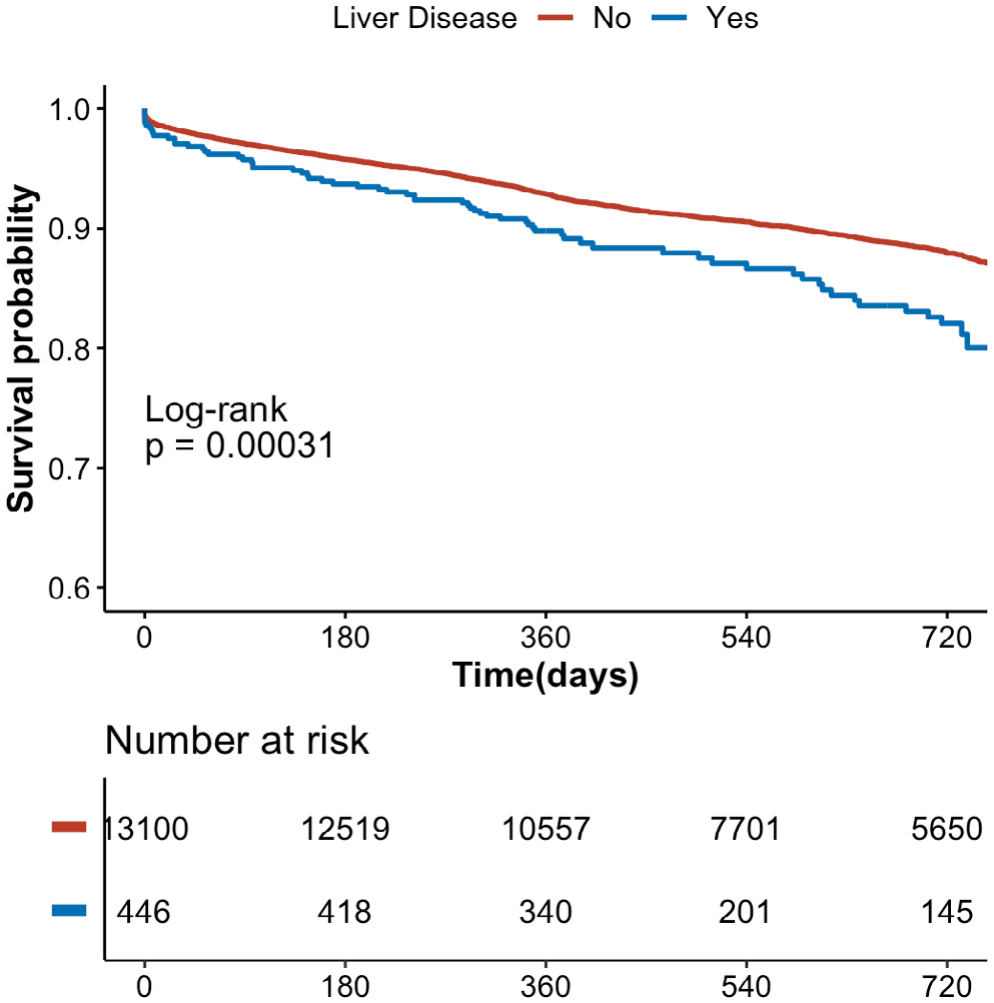
Kaplan–Meier curves for the Primary endpoint of the study, stratified by Liver disease status. Numbers at risk are shown below the plot. The corresponding adjusted hazard ratio is reported in Figure 3.

After a median follow‐up of 690 days [IQR 365–735], a total of 1699 patients met the primary endpoint of the study. AF patients with LD had a higher rate of events for composite outcome, MACEs and major bleeding (Table 2). Survival curves for the primary endpoint of the study are reported in Figure 2 (see Figure S2 for the secondary endpoints). The results show a significantly higher incidence of events for individuals with LD (Log‐rank Mantel‐Cox *p* < .001).

**TABLE 2:** Outcome of the study according to liver disease.

	No liver disease *N* = 15,164	Liver disease *N* = 517	*p*
Primary endpoint			
Composite outcome, *n* (%)	1628 (12.2)	71 (15.8)	.**028**
Secondary endpoint			
All‐cause death, *n* (%)	1062 (7.6)	45 (9.6)	.117
MACEs, *n* (%)	1009 (7.4)	47 (10.1)	.**032**
Any major bleeding, *n* (%)	248 (1.8)	15 (3.3)	.**030**

*Note:* Bold values indicate the *p* values that were significant.

Abbreviation: MACE, major adverse cardiovascular events.

**FIGURE 3:**
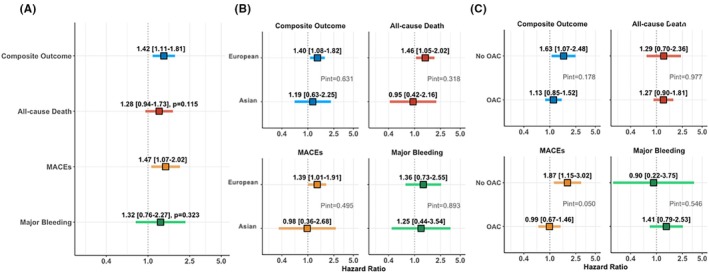
Multivariable Cox regression analyses for primary and secondary outcomes, with interaction by registry and oral anticoagulant use. (A) Overall population; (B) In the two registries; (C) OAC versus No OAC.

The results of Cox regression in the overall population are reported in Figure 3. After adjustments, AF patients with LD showed a higher risk of the primary endpoint (HR 1.42, 95% CI 1.11–1.81). Regarding the secondary exploratory outcomes, we found a higher risk of MACEs (HR 1.47, 95% CI 1.07–2.02), while no significant differences were observed for all‐cause death and major bleeding events.

Comparing the two registries, Asian patients with LD showed a lower percentage of events for all outcomes evaluated, except for major bleeding (Table S4).

After adjustments for possible confounders in the Cox regression analysis, no significant interaction effect was found between LD and the registries, that is, there were no significant ethnic (i.e., European vs. Asian) differences (Figure 3).

### 3.3 Comparison of OAC management

Outcome of patients according to OAC therapy are reported in Table S5. LD patients not treated with OAC showed a higher percentage of events for the composite outcome and MACEs (*p* = .003 and <.001, respectively). No differences were found for all‐cause death and major bleeding (*p* = .340 and *p* = .774, respectively).

After adjustments, the interaction analysis did not reveal a significant interaction effect between LD and OAC therapy on the modulation of the primary endpoint risk of the study (*p* = .178, [Figure 3]). Regarding the secondary exploratory outcomes, we found a significant interaction effect for the occurrence of MACEs (*p*_int_ = .050): in particular, patients with LD not treated with OAC showed a higher risk of the MACEs [HR 1.87, 95% CI 1.15–3.02] compared to patients treated with OAC [HR .99, 95% CI .67–1.46]. No significant interaction effect was evident for the remaining secondary endpoints.

Table S6 shows the percentages of adverse outcomes in the population taking NOACs versus VKAs, showing no difference between the two populations. These results were also confirmed at the interaction analysis (Figure S3).

## 4 DISCUSSION

In this analysis comparing two large prospective European and Asian AF registries, our main results are as follows: (i) in AF patients, LD increase the risk of a composite outcome of all‐cause death, any TE and any ACS, as well as the risk of MACEs, with no difference between European and Asian AF patients; (ii) LD had a significant impact on pharmacological treatment, causing a possible undertreatment for OAC therapy, with different trends among the two registries; and (iii) among LD patients with AF, OAC therapy did not increase the risk of bleeding and may have an effect on reducing MACEs, with no differences comparing NOACs versus VKAs.

To our knowledge, this is the first study reporting and comparing the association between LD and cardiovascular outcomes in two real‐world large cohorts of European and Asian AF patients. Not surprisingly, LD patients were found to be at higher risk of adverse clinical events. Indeed, these results are consistent with the previous literature. In the general population, LD has been previously found to be associated with a higher risk of major cardiovascular events.^16^, ^17^ Decreased endogenous anticoagulants and high levels of circulating procoagulants in patients with LD have been associated with an increased thrombotic risk.^18^ Similarly, the liver synthesizes most of the coagulation factors, exposing patients with a compromised liver function to a higher risk of bleeding.^18^ Hence, patients with LD, irrespective of the presence of AF, exhibit an imbalance between thrombotic and bleeding risks, which can alternatively exceed one at a time, the so‐called ‘coagulopathy of chronic liver disease’.^10^ Indeed, in a large Italian cohort of AF patients, those with LD were both at a significantly higher risk of thromboembolic and major bleeding events.^19^, ^20^ In our study, patients with LD showed a higher incidence of major bleeding events, which was not confirmed after adjustments for possible confounders and covariates. Nevertheless, residual confounding related to unmeasured factors such as liver function parameters, nutritional status or concomitant medications cannot be excluded and may have influenced the observed associations.

Etiologies of LD differ among European and Asian individuals.^21^ In particular, in Asia, higher prevalence rates of viral hepatitis and lower levels of alcohol and metabolic LD are reported.^22^ Comparing the cohorts of patients from our two registries, we found that Asian patients had a lower incidence of adverse outcomes. However, after taking account for important covariates, we found that LD did not have a different impact on the risk of cardiovascular events in patients from the two registries. These results suggest that, although the etiologies behind the LD may differ, the final common step of liver dysfunction resulting in an unbalanced control of thrombotic pathways in AF patients is detrimental in a similar way among individuals from both Europe and Asia.

Prior observations suggest that, even though patients with LD have a high‐risk thrombotic profile, ‘liver impairment’ may be associated with undertreatment with OAC therapy in AF patients.^9^ Our analysis further confirms these findings. This trend may have several explanations. First, as previously mentioned, LD patients are exposed to a higher risk of bleeding, and therefore, the decision to prescribe an OAC may be more challenging for many clinicians. Second, an optimal target for INR in LD individuals has not been validated (the standard target is 2.0–3.0). Moreover, LD patients have been found to have lower time in therapeutic range, thus limiting the use of VKAs in such recipients.^23^, ^24^ Third, patients with LD were substantially excluded from the four RCTs that validated the use of NOACs, and these drugs are contraindicated in case of a severe liver impairment (Child‐Pugh C for Apixaban, Edoxaban and Dabigatran, Child‐Pugh B for Rivaroxaban).^24^

Not surprisingly, the trend of underprescribing OAC therapy was particularly evident in European patients. The fact that the European registry began the enrolment in 2013, 2 years earlier than the Asian one, may be one of the explanations and may possibly influence the results of our analysis. Indeed, one very recent paper described how in US patients with cirrhosis from 2012 to 2019, the prescription of OAC increased from 39.4% to 49.0%, with decreasing use of VKAs and increasing use of NOACs.^25^

So, why has the use of OAC therapy increased in LD patients in recent years? As previously mentioned, one of the main concerns was the higher risk of bleeding associated with these patients. In recent years, our understanding of the issue has consistently improved, thanks to data derived from observational studies and meta‐analysis. In an article by Kuo et al.^26^ published in 2017 and held in Taiwan, in AF patients with LD at high risk of bleeding, there was no significant difference in intracranial bleeding in OAC‐treated patients (HR 1.27, 95% CI .82–1.95), with a reduction in stroke risk (HR .76, 95% CI .58–.99). Similarly, in a meta‐analysis published in 2019, the use of anticoagulation was not significantly associated with a higher risk of bleeding (HR 1.45, 95% CI .96–2.17), but it significantly reduced the risk of stroke (HR .58, 95% CI .35–.96).^27^ However, both these studies were conducted in an Asian prevalent population.

Consistent with the previous literature, our cohort study showed that OAC therapy did not increase the risk of major bleeding and may have a role in reducing the risk of MACEs. These findings may have significant clinical correlations: in fact, they suggest that OAC therapy may be beneficial for patients with LD, helping to reduce the high thrombotic pattern without a significant detrimental effect in terms of bleeding. Therefore, clinicians should not limit the prescription of OAC therapy among AF patients with LD when it is not clearly contraindicated.

Lastly, we showed that LD also significantly influences the management of symptoms in AF patients. Better symptom control with rhythm or rate control strategy is one of the cornerstones of the evidence‐based Atrial fibrillation Better Care (ABC) pathway^28^, ^29^, ^30^, ^31^ which is proposed as a tool to streamline the application of an integrated care approach in AF patients, together with the overall need of optimizing the long‐term management of LD.

Many AADs are metabolized in the liver and their dose may require some adjustment in the presence of LD. Moreover, Amiodarone and Dronedarone may be themselves causes of liver toxicity.^32^ Therefore, it is not surprising that we found that LD patients were less prescribed AADs. This trend was particularly true for Asian patients. As a matter of fact, the most feared complication of Amiodarone therapy is pulmonary toxicity. This complication was estimated to affect 2% of patients treated with Amiodarone in the follow up.^33^ However, Yamada et al. found a higher cumulative incidence of 10.6% at 5 years in a Japanese population.^34^

Similarly, LD influenced the prescription of Digoxin in AF patients, maybe because this drug has a renal prevalent metabolism. Again, we found a significant interaction effect between LD disease and the registries. Indeed, the lower probability of being prescribed digoxin for Asian patients may have several explanations. First, a recent study reported that digoxin was associated with a higher risk of a composite outcome of all‐cause death and thromboembolism [OR: 1.71; 95% CI: 1.32–2.22] in an AF cohort of elderly Chinese patients.^35^ Second, extensive use of herbal drugs in Asia may contribute to influencing the prescription of this drug.^36^

Overall, our findings highlight the detrimental role of LD in a contemporary cohort of European and Asian patients. OAC therapy, including both NOACs and VKAs, in the setting of LD appears to be safe and confers beneficial effects in terms of reducing the high thrombotic burden in these patients. However, the proportion of patients with LD in our cohort was relatively low, and no information on LD severity was available. Therefore, caution is warranted when extrapolating these findings to the overall AF population, particularly to patients with advanced or decompensated liver disease, who may be underrepresented in our study.

### 4.1 Study limitations

Our study has some limitations that should be acknowledged. Our data are derived from retrospective registries and, given the observational nature of our analysis, possible bias may be present in the interpretation of our findings. Furthermore, no formal a priori sample size calculation was performed, and the study population was determined by consecutive patient enrolment rather than by predefined power assumptions. Second, although multivariate analyses were performed to adjust for measured confounders, residual confounding remains possible due to the observational nature of the study. In particular, detailed information on liver function parameters, nutritional status and concomitant medications was not systematically collected and could not be accounted for in the analyses, potentially influencing the results of the analysis. Given the overall number of LD patients in our cohort, we may have reduced power to evaluate differences between groups. The secondary endpoint assessed in our analysis should be interpreted with caution and regarded as exploratory and hypothesis‐generating in nature. In addition, the relatively low proportion of patients with LD and the absence of predefined diagnostic algorithms or centralized adjudication, and the lack of information on disease severity limit the generalizability of our findings, particularly to patients with advanced or decompensated LD.

Our cohorts of patients are representative of those recruited from European and Asian countries; therefore, the results observed may not be entirely representative of the overall AF population. Moreover, we acknowledge that the differences found between the two registries may be related to the registries themselves, rather than being completely driven by specific ethnic differences in clinical epidemiology^37^ and AF‐related complications.^38^ Although the two registries had different follow‐up durations, the use of time‐to‐event analyses (Kaplan–Meier and Cox regression models) allowed appropriate handling of variable follow‐up through censoring; however, the shorter follow‐up in the Asian registry may have limited the number of observed events for some outcomes.

Lastly, data on time in therapeutic range and NOAC dosing were incomplete and therefore not included in the analyses to avoid potential bias, while information on anticoagulant plasma concentration monitoring was not available.

## 5 CONCLUSIONS

In this large contemporary cohort of European and Asian AF patients, the presence of LD was associated with a higher risk of adverse clinical outcomes. Among others, the presence of LD in AF is associated with a lower prescription rate of OAC, particularly among European patients. OAC therapy was not associated with a higher risk of bleeding, while a potential benefit may exist regarding the reduction of MACEs.

## AUTHOR CONTRIBUTIONS

DAM, TB, MP and GYHL conceived and designed the analysis. DAM and MP analysed data and drafted the manuscript. TB, BC, GB, AS, HFT, TFC, GB and GYHL revised the manuscript and gave relevant intellectual contributions. All authors read and approved the final manuscript.

## CONFLICT OF INTEREST STATEMENT

GFR reports consultancy for Boehringer Ingelheim and an educational grant from Anthos, outside the submitted work. No fees are directly received personally. GB is the Principal Investigator of the ARISTOTELES project (ApplyingARtificial Intelligence to define clinical trajectorieS for personalized predicTiOn and early deTEction of comorbidity and muLtimorbidity pattErnS) that received funding from the European Union within the Horizon 2020 research and innovation program (Grant N. 101080189) and reports small speaker fees from Bayer, Boehringer Ingelheim, Boston, BMS, Daiichi, Sanofi and Janssen outside the submitted work. GYHL: Consultant and speaker for BMS/Pfizer, Boehringer Ingelheim, Daiichi‐Sankyo, Anthos. No fees are received personally. He is a National Institute for Health and Care Research (NIHR) Senior Investigator and co‐PI of the AFFIRMO project on multimorbidity in AF (grant agreement No 899871), TARGET project on digital twins for personalized management of atrial fibrillation and stroke (grant agreement No 101136244) and ARISTOTELES project on artificial intelligence for management of chronic long term conditions (grant agreement No 101080189), which are all funded by the EU's Horizon Europe Research & Innovation programme. MP: Italian national Principal Investigator of the AFFIRMO project on multimorbidity in atrial fibrillation, which has received funding from the European Union's Horizon 2020 research and innovation program under grant agreement No 899871. The other authors do not have conflict of interests to report.

## Supporting information


Appendix S1.

## Data Availability

The data that support the findings of this study are available from the corresponding author upon reasonable request.
