# Supplementary material for: Liver disease in management and outcomes of European and Asian patients with atrial fibrillation: A report from two observational prospective registries
Source: Eur J Clin Invest. 2026 Mar 26;56(4):e70193. doi: 10.1111/eci.70193 (PMC13019421; doi:10.1111/eci.70193)

**Liver Disease in Management and Outcomes of European and Asian Patients with Atrial Fibrillation: A Report from Two Observational Prospective Registries**

**Supplementary Materials**

**TABLES**

**Supplementary Table 1.** Baseline characteristic of patient with Liver disease in the two registries.

|  | **EORP**  **N = 306 (59%)** | **APHRS**  **N = 211 (41%)** | **P** |
| --- | --- | --- | --- |
| **Age (years) (median [IQR])** | 68.00 [61.00, 75.00] | 70.00 [63.00, 76.00] | 0.038 |
| **Female, n (%)** | 121 (39.5) | 71 (33.6) | 0.204 |
| **BMI (median [IQR])** | 27.35 [24.20, 30.90] | 24.85 [22.50, 27.63] | **<0.001** |
| **Cardiovascular disease** |  |  |  |
| Hypertension, n (%) | 184 (61.1) | 143 (67.8) | 0.148 |
| Diabetes mellitus, n (%) | 94 (31.1) | 81 (38.6) | 0.098 |
| Dyslipidemia, n (%) | 152 (52.1) | 97 (46.4) | 0.248 |
| Coronary artery disease, n (%) | 98 (35.9) | 42 (20.1) | **<0.001** |
| Heart Failure, n (%) | 199 (65.7) | 59 (29.2) | **<0.001** |
| Previous TE events, n (%) | 33 (10.9) | 36 (17.3) | 0.053 |
| Peripheral vascular disease, n (%) | 44 (15.2) | 4 (1.9) | **<0.001** |
| **Comorbidities** |  |  |  |
| CKD, n (%) | 75 (24.8) | 31 (14.8) | **0.008** |
| CrCl CG (median [IQR]) | 68.34 [49.95, 92.76] | 62.50 [46.74, 81.51] | **0.040** |
| Malignancy (current+prior), n (%) | 21 (7.1) | 30 (14.3) | **0.013** |
| Previous hemorrhagic events, n (%) | 23 (7.7) | 22 (10.5) | 0.358 |
| Anemia, n (%) | 46 (15.1) | 26 (12.3) | 0.447 |
| **AF type, n (%)** |  |  | **<0.001** |
| First diagnosed | 38 (12.7) | 17 (8.1) |  |
| Paroxysmal | 58 (19.4) | 68 (32.2) |  |
| Persistent | 53 (17.7) | 45 (21.3) |  |
| Long-standing persistent | 15 (5.0) | 21 (10.0) |  |
| Permanent | 135 (45.2) | 60 (28.4) |  |
| **CHA2DS2-VASc (median [IQR])** | 3.00 [2.00, 5.00] | 3.00 [2.00, 5.00] | 0.195 |
| **HAS-BLED (median [IQR])** | 3.00 [2.00, 3.00] | 3.00 [2.00, 3.00] | 0.821 |
| **EHRA score 3-4, n (%)** | 90 (29.4) | 11 (5.2) | **<0.001** |

Legend: CG, Cockcroft-Gault; CKD, chronic kidney disease; CrCl, creatinine clearance; IQR, interquartile range; TE, thromboembolic events.

**Supplementary Table 2.** Pharmacological treatment in the overall population accordingly to Liver disease

|  | **No Liver Disease**  **N= 15164 (96.7%)** | **Liver Disease**  **N = 517 (3.3%)** | **P** |
| --- | --- | --- | --- |
| **Antiplatelet therapy, n (%)** | 2796 (18.5) | 115 (22.2) | **0.034** |
| **VKAs, n (%)** | 6246 (41.2) | 225 (43.5) | 0.315 |
| **NOACs, n (%)** | 6541 (43.2) | 185 (35.8) | **0.001** |
| **Any OAC, n (%)** | 12778 (84.3) | 410 (79.3) | **0.003** |
| **Any AADs, n (%)** | 3996 (26.5) | 104 (20.3) | **0.002** |
| **Class IC AADs, n (%)** | 1259 (8.3) | 24 (4.7) | **0.004** |
| **Class III AADs, n (%)** | 2788 (18.5 | 80 (15.6) | **0.116** |
| **Any rate control, n (%)** | 10833 (71.7) | 389 (76.3) | **0.027** |
| **ACE-inhibitors, n (%)** | 5140 (34.0) | 169 (33.2) | 0.735 |
| **ARBs, n (%)** | 3182 (21.1) | 107 (20.9) | 0.989 |
| **Beta-blockers, n (%)** | 9603 (63.6) | 327 (64.1) | 0.837 |
| **Aldosterone blocker, n (%)** | 2179 (14.4) | 123 (24.1) | **<0.001** |
| **Diuretics, n (%)** | 6405 (42.4) | 279 (54.6) | **<0.001** |
| **Digoxin, n (%)** | 1999 (13.2) | 123 (24.1) | **<0.001** |
| **Calcium channel blockers, n (%)** | 2787 (18.4) | 103 (20.2) | 0.347 |
| **Non-DHP CCB, n (%)** | 1163 (7.7) | 50 (9.8) | 0.096 |
| **Statin, n (%)** | 6161 (40.8) | 205 (40.0) | 0.764 |
| **Oral antidiabetics, n (%)** | 2313 (15.3) | 95 (18.6) | **0.050** |
| **Insulin, n (%)** | 681 (4.5) | 47 (9.2) | **<0.001** |

Legend: ARB, angiotensin receptor blocker; CCB, calcium channel blocker; NOAC, non-vitamin K antagonist; non-DHP, non-dihydropyridine; OAC, oral anticoagulant, VKA, vitamin K antagonist.

**Supplementary Table 3.** Pharmacological treatment of patients with Liver disease in the two registries.

|  | **EORP**  **N = 306** | **APHRS**  **N = 211** | **P** |
| --- | --- | --- | --- |
| **Antiplatelet therapy, n (%)** | 77 (25.2) | 38 (18.0) | 0.070 |
| **VKAs, n (%)** | 170 (55.6) | 55 (26.1) | **<0.001** |
| **NOACs, n (%)** | 65 (21.2) | 120 (56.9) | **<0.001** |
| **Any OAC, n (%)** | 235 (76.8) | 175 (82.9) | 0.113 |
| **Any AADs, n (%)** | 76 (25.1) | 28 (13.4) | **0.002** |
| **Class IC AAD, n (%)** | 11 (3.6) | 13 (6.2) | 0.254 |
| **Class III AAD, n (%)** | 65 (21.5) | 15 (7.2) | **<0.001** |
| **Any rate control, n (%)** | 245 (80.9) | 144 (69.6) | **0.005** |
| **ACE-inhibitors, n (%)** | 136 (45.2) | 33 (15.9) | **<0.001** |
| **ARBs, n (%)** | 54 (17.9) | 53 (25.4) | 0.053 |
| **Beta-blockers, n (%)** | 207 (68.3) | 120 (58.0) | **0.022** |
| **Aldosterone blocker, n (%)** | 107 (35.5) | 16 (7.7) | **<0.001** |
| **Diuretics, n (%)** | 220 (72.6) | 59 (28.4) | **<0.001** |
| **Digoxin, n (%)** | 90 (29.9) | 33 (15.8) | **<0.001** |
| **Calcium channel blockers, n (%)** | 43 (14.3) | 60 (28.7) | **<0.001** |
| **Non-DHP CCB, n (%)** | 17 (5.6) | 33 (15.8) | **<0.001** |
| **Statin, n (%)** | 110 (36.2) | 95 (45.7) | **0.039** |
| **Oral antidiabetics, n (%)** | 53 (17.5) | 42 (20.2) | 0.512 |
| **Insulin, n (%)** | 39 (12.9) | 8 (3.8) | **0.001** |

Legend: AAD, anti-arrhythmic drug; ARB, angiotensin receptor blocker; CCB, calcium channel blocker; NOAC, non-vitamin K antagonist; non-DHP, non-dihydropyridine; OAC, oral anticoagulant, VKA, vitamin K antagonist.

**Supplementary Table 4.** Outcome of the study among patients with Liver disease in the two registries.

|  | **EORP**  **N = 306** | **APHRS**  **N = 211** | **P** |
| --- | --- | --- | --- |
| **Primary Endpoint** |  |  |  |
| Composite outcome, n (%) | 61 (22.6) | 10 (5.6) | **<0.001** |
| **Secondary Endpoint** |  |  |  |
| All-cause death, n (%) | 39 (13.8) | 6 (3.3) | **<0.001** |
| MACEs, n (%) | 43 (15.1) | 4 (2.2) | **<0.001** |
| Any major bleeding, n (%) | 11 (4.0) | 4 (2.3) | 0.462 |

Legend MACE, major adverse cardiovascular events.

**Supplementary Table 5.** Outcome of the study among patients with Liver disease according to OAC prescription

|  | **No OAC**  **N = 107** | **OAC**  **N = 410** | **P** |
| --- | --- | --- | --- |
| **Primary Endpoint** |  |  |  |
| Composite outcome, n (%) | 24 (26.7) | 47 (13.1) | **<0.003** |
| **Secondary Endpoint** |  |  |  |
| All-cause death, n (%) | 12 (12.8) | 33 (8.8) | 0.340 |
| MACEs, n (%) | 19 (20.4) | 28 (7.5) | **<0.001** |
| Any major bleeding, n (%) | 2 (2.3) | 13 (3.6) | 0.774 |

Legend MACE, major adverse cardiovascular events.

**Supplementary Table 6.** Outcome of the study among patients with Liver disease taking NOAC vs VKA

|  | **VKA**  **N = 225** | **NOAC**  **N = 185** | **P** |
| --- | --- | --- | --- |
| **Primary Endpoint** |  |  |  |
| Composite outcome, n (%) | 30 (15.0) | 17 (10.7) | 0.296 |
| **Secondary Endpoint** |  |  |  |
| All-cause death, n (%) | 22 (10.6) | 11 (6.7) | 0.255 |
| MACEs, n (%) | 20 (9.5) | 8 (5.0) | 0.148 |
| Any major bleeding, n (%) | 9 (4.4) | 4 (2.5) | 0.504 |

Legend MACE, major adverse cardiovascular events.

**FIGURES**

**Supplementary Figure 1.** Impact of Liver disease on the prescription of rhythm and rate control drugs.

Panel A: Overall Population; Panel B: European vs Asian


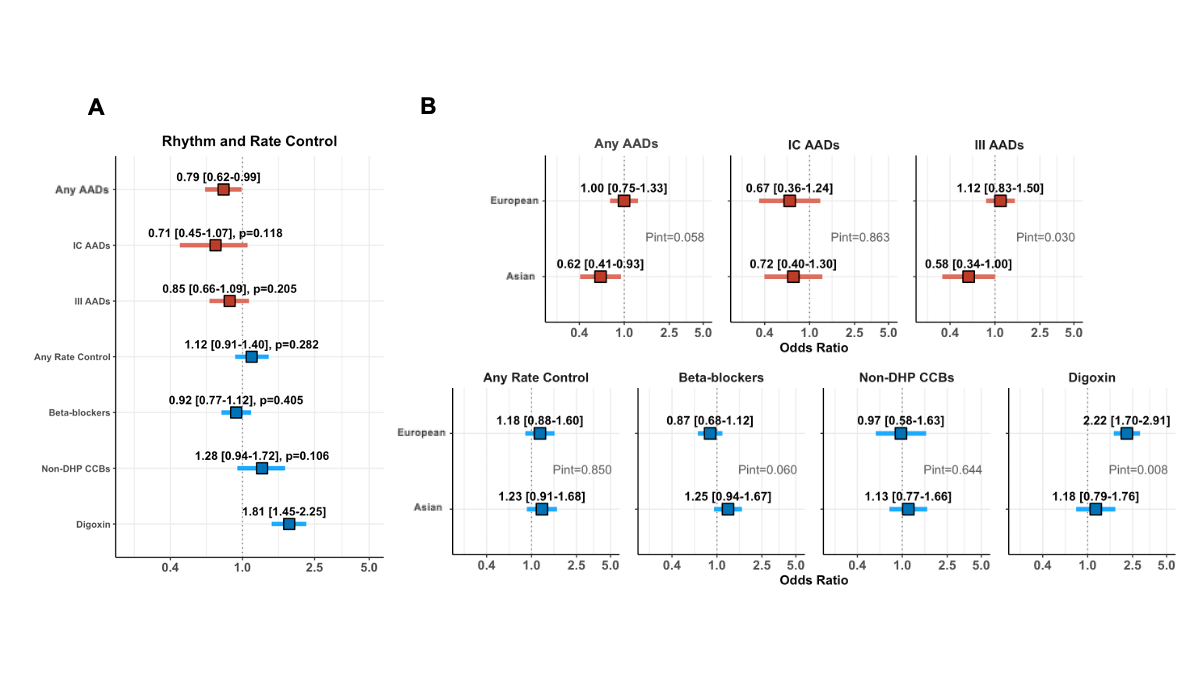


Legend AAD, antiarrhythmic drug; CCB, calcium channel blockers; non-DHP, non-dihydropyridine

**Supplementary Figure 2.** Kaplan-Meier curves for secondary endpoints of the study.

Panel A: All-cause Death; Panel B: MACEs; Panel C: Any major bleeding.


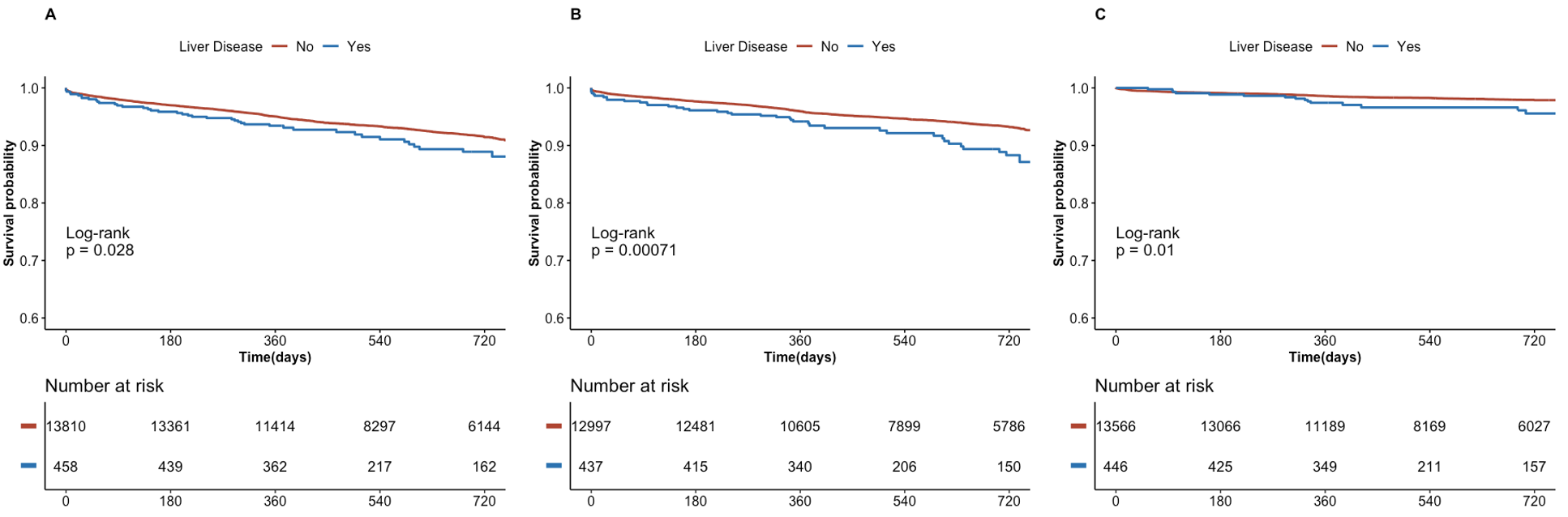


Legend. Numbers at risk are shown below each plot. The relative adjusted Hazard ratios and 95% CI are reported in Figure 3 in the main paper.

**Supplementary Figure 3.** Interaction analysis for comparison of NOAC vs VKA.


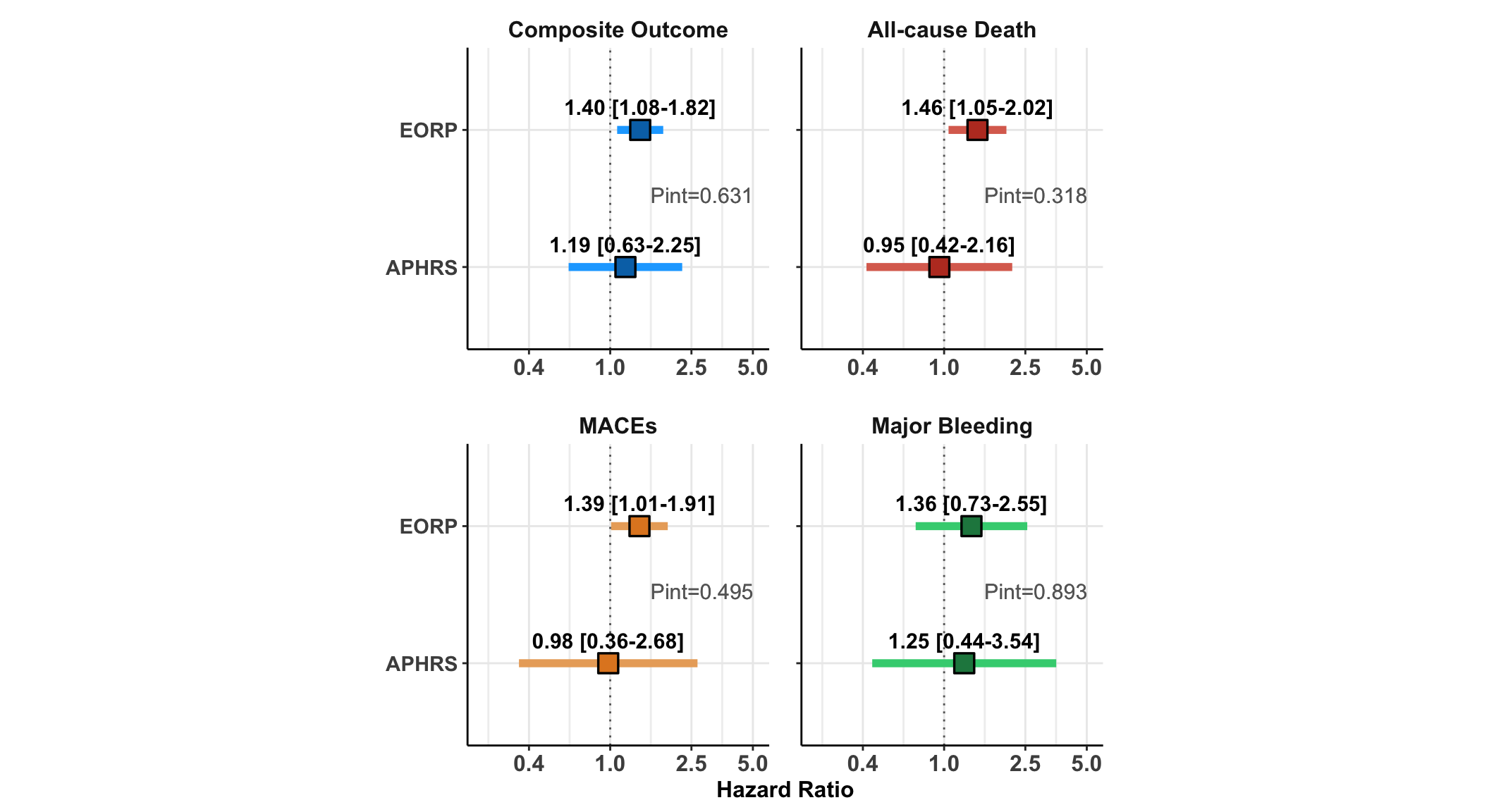

Supplement: Supplementary file 1 — Appendix S1. [file ECI-56-e70193-s001.docx]
